# Movable Kidney

**Published:** 1905-01-28

**Authors:** 


### MOVABLE KIDNEY.

*To the Editor of* THE HOSPITAL.

DEAR SIR,—By a strange coincidence, I received on the same day that I read in your issue of the 14th inst. the notice of Sir Frederick Treves's remarks in the *Practitioner* on "Movable Kidney" the enclosed memo. The patient on whom the nephropexy was performed two and a half years since had worn, without avail, a carefully adjusted belt for some years, and was, when operated upon, in a most serious condition from a large movable kidney. As you see, the present report is "that she was never so well in all her life." I have known many cases in which symptoms which have lasted for years have been completely relieved by the operation. It is quite true that successful fixation of the organ does not always follow, but when it does I consider the results are as beneficial as can be desired. There are reasons other than the mere temporary relief of the symptoms why fixation of a floating kidney should be carried out. These I do not enter upon here, but I do desire in some degree to controvert what I consider to be the far too sweeping condemnation of the operation that has appeared in your columns. I quite agree, however, with your conclusion that the operation "should be the last, and not the first, resource."

Enclosing my card, I remain, yours faithfully,

OPERATING SURGEON.

